# 1-(Prop-2-yn­yl)indoline-2,3-dione

**DOI:** 10.1107/S1600536814003973

**Published:** 2014-02-26

**Authors:** Fatima-Zahrae Qachchachi, Fouad Ouazzani Chahdi, Houria Misbahi, Michael Bodensteiner, Lahcen El Ammari

**Affiliations:** a Laboratoire de Chimie Organique Appliquée, Université Sidi Mohamed Ben Abdallah, Faculté des Sciences et Techniques, Route d’Immouzzer, BP 2202 Fès, Morocco; b Institut National des Plantes Médicinales et Aromatiques, Université Sidi Mohamed Ben Abdallah, BP 2202 Fès, Morocco; c X-Ray Structure Analysis Unit, University of Regensburg, D-93053 Regensburg, Germany; d Laboratoire de Chimie du Solide Appliquée, Faculté des Sciences, Université Mohammed V-Agdal, Avenue Ibn Battouta, BP 1014, Rabat, Morocco

## Abstract

The structure of the title compound, C_11_H_7_NO_2_, is isotypic to that of its homologue, 1-octylindoline-2,3-dione [Qachchachi *et al.* (2013 ▶). *Acta Cryst.* E**69**, o1801]. The indoline ring and the two carbonyl O atoms are approximately coplanar, the largest deviation from the mean plane being 0.021 (1) Å for one of the O atoms. The mean plane through the fused ring system is nearly perpendicular to the propynyl group, as indicated by the N—C—C—C torsion angle of 77.9 (1)°. In the crystal, mol­ecules are linked by C—H⋯O hydrogen bonds and π–π inter­actions between benzene rings [inter­centroid distance = 3.5630 (10) Å], forming a three-dimensional structure.

## Related literature   

For the biological activity of indoline derivatives, see: Malhotra *et al.* (2011 ▶); Ramachandran (2011 ▶); Smitha *et al.* (2008 ▶). For the structure of 1-octylindoline-2,3-dione, see: Qachchachi *et al.* (2013 ▶).
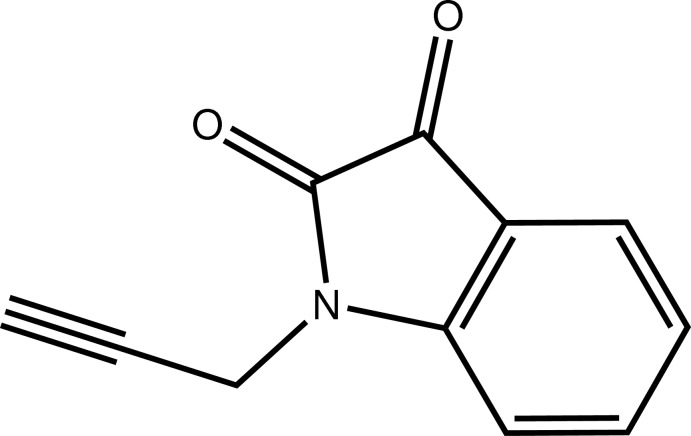


## Experimental   

### 

#### Crystal data  

C_11_H_7_NO_2_
*M* *_r_* = 185.18
Triclinic, 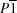

*a* = 7.0939 (7) Å
*b* = 7.9452 (7) Å
*c* = 8.5658 (6) Å
α = 80.464 (7)°
β = 85.760 (7)°
γ = 63.881 (9)°
*V* = 427.50 (6) Å^3^
*Z* = 2
Cu *K*α radiation
μ = 0.83 mm^−1^
*T* = 123 K
0.15 × 0.13 × 0.02 mm

#### Data collection  

Agilent SuperNova (Single source at offset, Atlas) diffractometer
Absorption correction: analytical (Clark & Reid, 1995 ▶) *T*_min_ = 0.910, *T*_max_ = 0.981
3081 measured reflections
1627 independent reflections
1438 reflections with *I* > 2σ(*I*)
*R*_int_ = 0.029

#### Refinement  

*R*[*F* ^2^ > 2σ(*F* ^2^)] = 0.037
*wR*(*F* ^2^) = 0.094
*S* = 1.07
1627 reflections
127 parameters
H-atom parameters constrained
Δρ_max_ = 0.20 e Å^−3^
Δρ_min_ = −0.22 e Å^−3^

### 

Data collection: *CrysAlis PRO* (Agilent, 2013 ▶); cell refinement: *CrysAlis PRO*; data reduction: *CrysAlis PRO*; program(s) used to solve structure: *SHELXS97* (Sheldrick, 2008 ▶); program(s) used to refine structure: *SHELXL97* (Sheldrick, 2008 ▶); molecular graphics: *ORTEP-3 for Windows* (Farrugia, 2012 ▶); software used to prepare material for publication: *WinGX* (Farrugia, 2012 ▶) and *publCIF* (Westrip, 2010 ▶).

## Supplementary Material

Crystal structure: contains datablock(s) I. DOI: 10.1107/S1600536814003973/tk5296sup1.cif

Structure factors: contains datablock(s) I. DOI: 10.1107/S1600536814003973/tk5296Isup2.hkl

Supporting information file. DOI: 10.1107/S1600536814003973/tk5296Isup3.cml

CCDC reference:

Additional supporting information:  crystallographic information; 3D view; checkCIF report

## Figures and Tables

**Table 1:** Hydrogen-bond geometry (Å, °)

*D*—H⋯*A*	*D*—H	H⋯*A*	*D*⋯*A*	*D*—H⋯*A*
C5—H5⋯O1^i^	0.95	2.62	3.3901 (17)	139
C9—H9*A*⋯O1^ii^	0.99	2.61	3.4747 (18)	146
C9—H9*B*⋯O2^iii^	0.99	2.37	3.2987 (17)	156

Symmetry codes: (i) 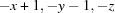; (ii) 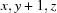; (iii) 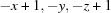.

**Fig. 1.**
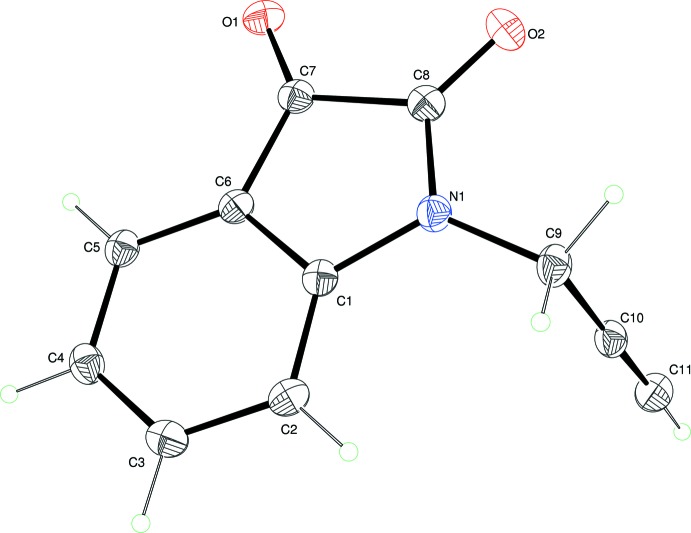
Molecular plot the title compound with the atom-labelling scheme. Displacement ellipsoids are drawn at the 50% probability level.

**Fig. 2.**
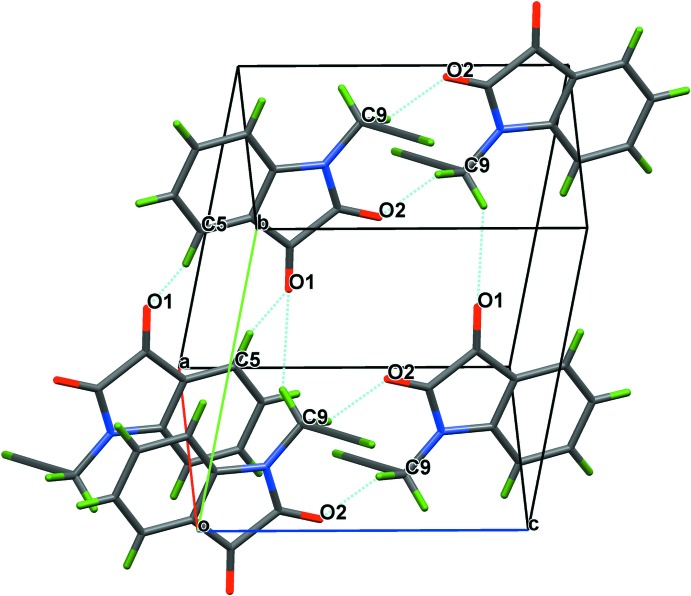
Intermolecular hydrogen interactions in the title compound. Hydrogen bonds are shown as dashed lines.

## supplementary crystallographic information

### 2. Structural commentary

Isatin (1*H*-indole-2,3-dione) is a synthetically versatile substrate,used for the synthesis of a large variety of heterocyclic compounds, such as indoles and quinolines, and as a raw material for drug synthesis. Isatin has also been found in mammalian tissues, and its function as a modulator of biochemical processes has been the subject of several discussions. Isatin and its derivatives have aroused great attention in recent years due to their wide variety of biological activities, relevant to application as insecticides and fungicides and in a broad range of drug therapies, including as anti-cancer agents, anti-biotics and anti-depressants (Malhotra *et al.*, 2011; Ramachandran, 2011; Smitha *et al.*, 2008). In our work, we are inter­ested in developing a new isatin derivative with the addition of alkyl halides to explore other applications (Scheme 1).

The molecule of title compound is build up from a fused five- and six-membered rings linked, to the propynyl chain and to two carbonyl group O atoms as shown in Fig. 1. The indoline ring and the two carbonyl O atoms are nearly co-planar, with the largest deviation from the mean plane being 0.021 (1) Å for the O1 atom. The fused ring system plan is nearly perpendicular to the the propynyl chain as indicated by N1—C9—C10—C11 torsion angle of 77.9 (1)°. The structure of the title compound is similar to that of its homologue 1-octylindoline-2,3-dione (Qachchachi *et al.*, 2013).

In the crystal, the molecules are linked by C—H···O1 hydrogen bonds, Table 1 and Fig. 2, to form layers in hte bc plane. Layers are connected by π—π inter­actions between centrosymmetrically related benzene rings [3.5630 (10) Å; symmetry operation: -*x*, -*y*, -*z*] in the way to build a three-dimensional network.

### 5. Synthesis and crystallization

To a solution of isatin (0.5 g, 3.4 mmol) dissolved in DMF (30 ml) was added potassium carbonate (0.61 g, 4.4 mmol), a catalytic qu­antity of tetra-n- butyl­ammonium (0.1 g, 0.4 mmol) and 3-bromo­prop-1-yne (0.3 ml, 3.7 mmol). The mixture was stirred for 48 h; the reaction was monitored by thin layer chromatography. The mixture was filtered and the solvent removed under vacuum. The solid obtained was recrystallized from ethanol to afford the title compound as red crystals in 88% yield (M.pt: 423 K).

### 6. Refinement

All H atoms could be located in a difference Fourier map. However, they were placed in calculated positions with C—H = 0.95 Å (aromatic, acetyl­enic) and C—H = 0.99 Å (methyl­ene), and refined as riding on their parent atoms with *U*_iso_(H) = 1.2 *U*_eq_ (aromatic, acetyl­enic and methyl­ene).

### Crystal data

**Table:**

C_11_H_7_NO_2_	*Z* = 2
*M**_r_* = 185.18	*F*(000) = 192
Triclinic, *P*1	*D*_x_ = 1.439 Mg m^−^^3^
Hall symbol: -P 1	Melting point: 423 K
*a* = 7.0939 (7) Å	Cu *K*α radiation, λ = 1.54184 Å
*b* = 7.9452 (7) Å	Cell parameters from 1851 reflections
*c* = 8.5658 (6) Å	θ = 5.2–73.3°
α = 80.464 (7)°	µ = 0.83 mm^−^^1^
β = 85.760 (7)°	*T* = 123 K
γ = 63.881 (9)°	Plate, red
*V* = 427.50 (6) Å^3^	0.15 × 0.13 × 0.02 mm

### Data collection

**Table:**

Agilent SuperNova (Single source at offset, Atlas) diffractometer	1627 independent reflections
Radiation source: SuperNova (Cu) X-ray Source	1438 reflections with *I* > 2σ(*I*)
Mirror monochromator	*R*_int_ = 0.029
Detector resolution: 10.3546 pixels mm^-1^	θ_max_ = 73.5°, θ_min_ = 5.2°
ω scans	*h* = −8→8
Absorption correction: analytical (Clark & Reid, 1995)	*k* = −9→9
*T*_min_ = 0.910, *T*_max_ = 0.981	*l* = −7→10
3081 measured reflections	

### Refinement

**Table:**

Refinement on *F*^2^	Primary atom site location: structure-invariant direct methods
Least-squares matrix: full	Secondary atom site location: difference Fourier map
*R*[*F*^2^ > 2σ(*F*^2^)] = 0.037	Hydrogen site location: inferred from neighbouring sites
*wR*(*F*^2^) = 0.094	H-atom parameters constrained
*S* = 1.07	*w* = 1/[σ^2^(*F*_o_^2^) + (0.0429*P*)^2^ + 0.0993*P*] where *P* = (*F*_o_^2^ + 2*F*_c_^2^)/3
1627 reflections	(Δ/σ)_max_ < 0.001
127 parameters	Δρ_max_ = 0.20 e Å^−^^3^
0 restraints	Δρ_min_ = −0.22 e Å^−^^3^

### Special details

**Table:**

Geometry. All e.s.d.'s (except the e.s.d. in the dihedral angle between two l.s. planes) are estimated using the full covariance matrix. The cell e.s.d.'s are taken into account individually in the estimation of e.s.d.'s in distances, angles and torsion angles; correlations between e.s.d.'s in cell parameters are only used when they are defined by crystal symmetry. An approximate (isotropic) treatment of cell e.s.d.'s is used for estimating e.s.d.'s involving l.s. planes.
Refinement. Refinement of *F*^2^ against all reflections. The weighted *R*-factor *wR* and goodness of fit *S* are based on *F*^2^, conventional *R*-factors *R* are based on *F*, with *F* set to zero for negative *F*^2^. The threshold expression of *F*^2^ > σ(*F*^2^) is used only for calculating *R*-factors(gt) *etc*. and is not relevant to the choice of reflections for refinement. *R*-factors based on *F*^2^ are statistically about twice as large as those based on *F*, and *R*- factors based on all data will be even larger.

### Fractional atomic coordinates and isotropic or equivalent isotropic displacement parameters (Å^2^)

**Table:**

	*x*	*y*	*z*	*U*_iso_*/*U*_eq_	
C1	0.25017 (19)	0.06257 (19)	0.05480 (16)	0.0180 (3)	
C2	0.1753 (2)	0.2210 (2)	−0.05932 (17)	0.0211 (3)	
H2	0.1510	0.3421	−0.0368	0.025*	
C3	0.1367 (2)	0.1959 (2)	−0.20994 (16)	0.0225 (3)	
H3	0.0847	0.3028	−0.2906	0.027*	
C4	0.1719 (2)	0.0202 (2)	−0.24516 (16)	0.0224 (3)	
H4	0.1440	0.0084	−0.3486	0.027*	
C5	0.2481 (2)	−0.1391 (2)	−0.12896 (16)	0.0204 (3)	
H5	0.2729	−0.2602	−0.1517	0.025*	
C6	0.2867 (2)	−0.11607 (19)	0.02066 (16)	0.0186 (3)	
C7	0.3621 (2)	−0.25174 (19)	0.16566 (16)	0.0201 (3)	
C8	0.3703 (2)	−0.1326 (2)	0.29023 (16)	0.0204 (3)	
C9	0.2676 (2)	0.2094 (2)	0.29379 (16)	0.0222 (3)	
H9A	0.2986	0.3039	0.2198	0.027*	
H9B	0.3644	0.1648	0.3849	0.027*	
C10	0.0499 (2)	0.29983 (19)	0.34996 (16)	0.0210 (3)	
C11	−0.1255 (2)	0.3711 (2)	0.39699 (17)	0.0249 (3)	
H11	−0.2657	0.4280	0.4346	0.030*	
N1	0.30354 (18)	0.04896 (16)	0.21361 (13)	0.0203 (3)	
O1	0.40885 (16)	−0.41925 (14)	0.19499 (12)	0.0263 (3)	
O2	0.42444 (16)	−0.19092 (15)	0.42712 (12)	0.0262 (3)	

### Atomic displacement parameters (Å^2^)

**Table:**

	*U*^11^	*U*^22^	*U*^33^	*U*^12^	*U*^13^	*U*^23^
C1	0.0119 (6)	0.0223 (6)	0.0210 (6)	−0.0082 (5)	0.0020 (5)	−0.0050 (5)
C2	0.0172 (6)	0.0193 (6)	0.0270 (7)	−0.0082 (5)	0.0014 (5)	−0.0040 (5)
C3	0.0177 (6)	0.0251 (7)	0.0230 (7)	−0.0093 (5)	0.0003 (5)	0.0010 (5)
C4	0.0196 (7)	0.0296 (7)	0.0192 (6)	−0.0116 (6)	0.0015 (5)	−0.0050 (5)
C5	0.0161 (6)	0.0230 (7)	0.0230 (6)	−0.0085 (5)	0.0023 (5)	−0.0066 (5)
C6	0.0143 (6)	0.0196 (6)	0.0218 (6)	−0.0073 (5)	0.0015 (5)	−0.0039 (5)
C7	0.0142 (6)	0.0222 (7)	0.0229 (6)	−0.0073 (5)	0.0008 (5)	−0.0033 (5)
C8	0.0150 (6)	0.0247 (7)	0.0223 (7)	−0.0096 (5)	0.0014 (5)	−0.0031 (5)
C9	0.0219 (7)	0.0248 (7)	0.0240 (7)	−0.0120 (6)	−0.0005 (5)	−0.0089 (5)
C10	0.0260 (7)	0.0204 (6)	0.0193 (6)	−0.0117 (6)	−0.0025 (5)	−0.0042 (5)
C11	0.0240 (7)	0.0240 (7)	0.0271 (7)	−0.0103 (6)	0.0003 (6)	−0.0057 (5)
N1	0.0196 (6)	0.0213 (6)	0.0204 (6)	−0.0085 (5)	−0.0003 (4)	−0.0051 (4)
O1	0.0261 (5)	0.0193 (5)	0.0307 (5)	−0.0079 (4)	−0.0018 (4)	−0.0012 (4)
O2	0.0266 (5)	0.0343 (6)	0.0197 (5)	−0.0153 (5)	−0.0027 (4)	−0.0016 (4)

### Geometric parameters (Å, º)

**Table:**

C1—C2	1.379 (2)	C6—C7	1.4622 (18)
C1—C6	1.4034 (18)	C7—O1	1.2072 (17)
C1—N1	1.4131 (17)	C7—C8	1.5589 (18)
C2—C3	1.402 (2)	C8—O2	1.2111 (18)
C2—H2	0.9500	C8—N1	1.3663 (18)
C3—C4	1.387 (2)	C9—N1	1.4631 (17)
C3—H3	0.9500	C9—C10	1.470 (2)
C4—C5	1.394 (2)	C9—H9A	0.9900
C4—H4	0.9500	C9—H9B	0.9900
C5—C6	1.3872 (19)	C10—C11	1.188 (2)
C5—H5	0.9500	C11—H11	0.9500
			
C2—C1—C6	121.15 (12)	O1—C7—C6	131.72 (13)
C2—C1—N1	128.42 (13)	O1—C7—C8	123.44 (12)
C6—C1—N1	110.42 (12)	C6—C7—C8	104.83 (11)
C1—C2—C3	117.21 (13)	O2—C8—N1	127.55 (13)
C1—C2—H2	121.4	O2—C8—C7	126.40 (13)
C3—C2—H2	121.4	N1—C8—C7	106.05 (11)
C4—C3—C2	122.21 (13)	N1—C9—C10	111.50 (11)
C4—C3—H3	118.9	N1—C9—H9A	109.3
C2—C3—H3	118.9	C10—C9—H9A	109.3
C3—C4—C5	120.08 (12)	N1—C9—H9B	109.3
C3—C4—H4	120.0	C10—C9—H9B	109.3
C5—C4—H4	120.0	H9A—C9—H9B	108.0
C6—C5—C4	118.27 (13)	C11—C10—C9	179.14 (16)
C6—C5—H5	120.9	C10—C11—H11	180.0
C4—C5—H5	120.9	C8—N1—C1	111.08 (11)
C5—C6—C1	121.08 (13)	C8—N1—C9	123.22 (12)
C5—C6—C7	131.32 (13)	C1—N1—C9	125.25 (12)
C1—C6—C7	107.60 (11)		
			
C6—C1—C2—C3	0.27 (19)	O1—C7—C8—O2	0.7 (2)
N1—C1—C2—C3	179.20 (13)	C6—C7—C8—O2	179.86 (13)
C1—C2—C3—C4	−0.2 (2)	O1—C7—C8—N1	−179.02 (13)
C2—C3—C4—C5	0.0 (2)	C6—C7—C8—N1	0.16 (14)
C3—C4—C5—C6	0.04 (19)	O2—C8—N1—C1	−178.89 (13)
C4—C5—C6—C1	0.04 (19)	C7—C8—N1—C1	0.81 (14)
C4—C5—C6—C7	178.89 (13)	O2—C8—N1—C9	−6.2 (2)
C2—C1—C6—C5	−0.20 (19)	C7—C8—N1—C9	173.50 (11)
N1—C1—C6—C5	−179.30 (12)	C2—C1—N1—C8	179.43 (13)
C2—C1—C6—C7	−179.30 (12)	C6—C1—N1—C8	−1.55 (16)
N1—C1—C6—C7	1.60 (15)	C2—C1—N1—C9	6.9 (2)
C5—C6—C7—O1	−0.9 (3)	C6—C1—N1—C9	−174.06 (12)
C1—C6—C7—O1	178.03 (14)	C10—C9—N1—C8	−90.49 (15)
C5—C6—C7—C8	179.97 (14)	C10—C9—N1—C1	81.16 (16)
C1—C6—C7—C8	−1.05 (14)		

### Hydrogen-bond geometry (Å, º)

**Table:**

*D*—H···*A*	*D*—H	H···*A*	*D*···*A*	*D*—H···*A*
C5—H5···O1^i^	0.95	2.62	3.3901 (17)	139
C9—H9*A*···O1^ii^	0.99	2.61	3.4747 (18)	146
C9—H9*B*···O2^iii^	0.99	2.37	3.2987 (17)	156

Symmetry codes: (i) −*x*+1, −*y*−1, −*z*; (ii) *x*, *y*+1, *z*; (iii) −*x*+1, −*y*, −*z*+1.
